# Performance Analyses of Photonic-Crystal Surface-Emitting Laser: Toward High-Speed Optical Communication

**DOI:** 10.1186/s11671-022-03728-x

**Published:** 2022-09-17

**Authors:** Chun-Yen Peng, Hao-Tien Cheng, Yu-Heng Hong, Wen-Cheng Hsu, Fu-He Hsiao, Tien-Chang Lu, Shu-Wei Chang, Shih-Chen Chen, Chao-Hsin Wu, Hao-Chung Kuo

**Affiliations:** 1 Semiconductor Research Center, Hon Hai Research Institute, Taipei, 11492 Taiwan; 2 Research Center for Applied Sciences, Academia Sinica, Nankang, Taipei, 11529 Taiwan; 3 Graduate Institute of Photonics and Optoelectronics, Graduate Institute of Electronics Engineering, and Graduate School of Advanced Technology, National Taiwan University, No. 1, Sec. 4, Roosevelt Rd., Taipei, 10617 Taiwan; 4 Department of Photonics and Institute of Electro-Optical Engineering, College of Electrical and Computer Engineering, National Yang Ming Chiao Tung University, Hsinchu, 30010 Taiwan

**Keywords:** Photonic-crystal surface-emitting laser, Small-signal analysis, Eye diagram, Bit-error-rate test, Optical communication

## Abstract

This study conducts comprehensive performance analyses of a commercial photonic-crystal surface-emitting laser (PCSEL) via small-signal measurement and the bit-error-rate test. Meanwhile, the radio frequency characteristics of the PCSEL are unveiled for the first time. Compared to the vertical-cavity surface-emitting lasers, the PCSEL shows great potential for a broader optical bandwidth that is benefited from the high optical-confinement factor. A maximum bandwidth of around 2.32 GHz is experimentally observed when the PCSEL was biased at 340 mA. Moreover, a theoretical calculation was applied to shed light on the characteristics of the small-signal measurement, providing a deep insight into the corresponding intrinsic response model. The signal transmission capability of the PCSEL was investigated as well. The maximum bit rate and corresponding rise time transmitted at 500 Mbps are 1.2 Gbps and 186.16 ps, respectively. Thus, a high-speed PCSEL can be realised with a shrunk form factor, serving as a promising candidate for the next-generation light sources in high-speed optical communication.

## Introduction

With the expansion of the Internet of Things (IoT) and low-earth-orbit (LEO) satellite technologies, interaction and integration among people and devices are flourishing. The technologies of end-to-end (E2E) or device-to-device (D2D) communications are essential for development. Moreover, such a satellite constellation can prosperously facilitate the connection of remote and rural communities behind, serving as a bridge to the digital divide. In the ancient world, humankind carries important messages through toilsome runners. People have also made various strategies over time, transferring data or messages across the distance. Among these strategies, visual techniques, such as smoke signals, beacon fires, hydraulic telegraphs, ship flags, and semaphore lines, can be the most direct way. These techniques transfer data or messages through visualisation, forming the earliest fashion of optical communication. In a contemporary society with the rapid development of semiconductor technology, people can adopt more appropriate light sources with expeditious needs, further revolutionising the development of the internet and wireless communication engineering. Within just a few centuries, we human beings have gotten into the Information Age, thus accelerating globalisation as well [1, 2].

Equipped with semiconductor technology, solid-state light-emitting devices for optical communication elicit dramatic changes, providing alternative solutions for wireless communication [3–7]. Nowadays, indoor wireless data exchanges reach up to 70% [8]. Accordingly, visible light communication (VLC) can be an expedient solution due to the proper combination of daily illumination, exploiting the indoor light-emitting diodes (LEDs), and optical communication [9]. However, VLC inevitably exhibits several shortcomings, such as the visual interference with constant light emission and short transmission range caused by the incoherent LEDs. Optical communication with near-infrared (NIR) light sources emerges to overcome the above-mentioned drawbacks [10, 11]. Moreover, an unsurpassed lasing action from the surface-emitting laser, namely the vertical-cavity surface-emitting laser (VCSEL), was demonstrated in 1988, pushing forward in various applications with its unique characteristics and performance [12–21]. VCSELs exhibit great benefits compared to the LEDs, such as a symmetric beam profile with a narrow spectrum, a low threshold current with high fiber coupling efficiency, and a high modulation bandwidth [22]. With contemporary semiconductor technology, such solid-state laser devices can provide undemanding integration and good compatibility with consumer electronics devices. Thus, VCSELs have become mainstream transmitters in optical communication [23].

Today, direct-modulated high-speed lasers dominate the optical-communication market. The most well-known lasers are distributed feedback (DFB) lasers and VCSELs. VCSELs are not ready yet for fabrication in the InP material system. Several reasons lie behind the inadequateness of InP-based VCSELs. First, distributed Bragg reflectors do not have a good material combination to meet the requirements of InP lattice matching, large refractive index difference, and high thermal conductivity [24, 25]. Second, the optical gain and the characteristic temperature of the gain medium for long-wavelength lasers, such as GaInAsP QWs, are poorer than GaAs and InGaAs QWs on GaAs. Third, there was no good way to confine the current to the centre of a mesa [26]. Hence, DFB lasers are the mainstream in long-wavelength transmission systems at 1.3 μm or 1.5 μm. However, it is difficult to fabricate and handle the DFB laser with a short cavity length (< 150 μm) using the cleaving method. Consequently, a large active region will restrict the modulation bandwidth. To achieve a high-speed DFB laser, a complex cavity design is required [27]. Fortunately, the active region of VCSELs can be effectively shrunk through the oxide layer in the GaAs material system [28]. The oxide layer can confine current paths and light distribution simultaneously. This feature overcomes the size issues of the active region, resulting in a modulation bandwidth beyond 30 GHz [29]. Nevertheless, for the conventional VCSEL structures, the lack of optical confinement factor in emission direction has become an obstacle, hindering the further development of modulation speed in recent years. The confinement factor of a high-speed VCSEL remains below 4% [30], which severely limits the improvement capability of a VCSEL bandwidth.

From the perspective of miniaturisation with better light-emitting performance, new concepts are thus in the acute stage with rapid development toward an ultra-compact optical device with various functionalities [31]. Auspiciously, a revolutionary semiconductor laser technology, the photonic-crystal surface-emitting laser (PCSEL), emerged over the past two decades [32–35]. A photonic crystal can be adopted as a lateral laser cavity via monolithic integration embedded in the epitaxial structures, further ameliorating the bulky form factor compared to the laser cavities in VCSEL structures. Laser light emission from such PCSELs exhibits excellent performance, such as a symmetric beam profile with narrow beam divergence and a narrower spectral width than that of VCSELs [36].

**Fig. 1:**
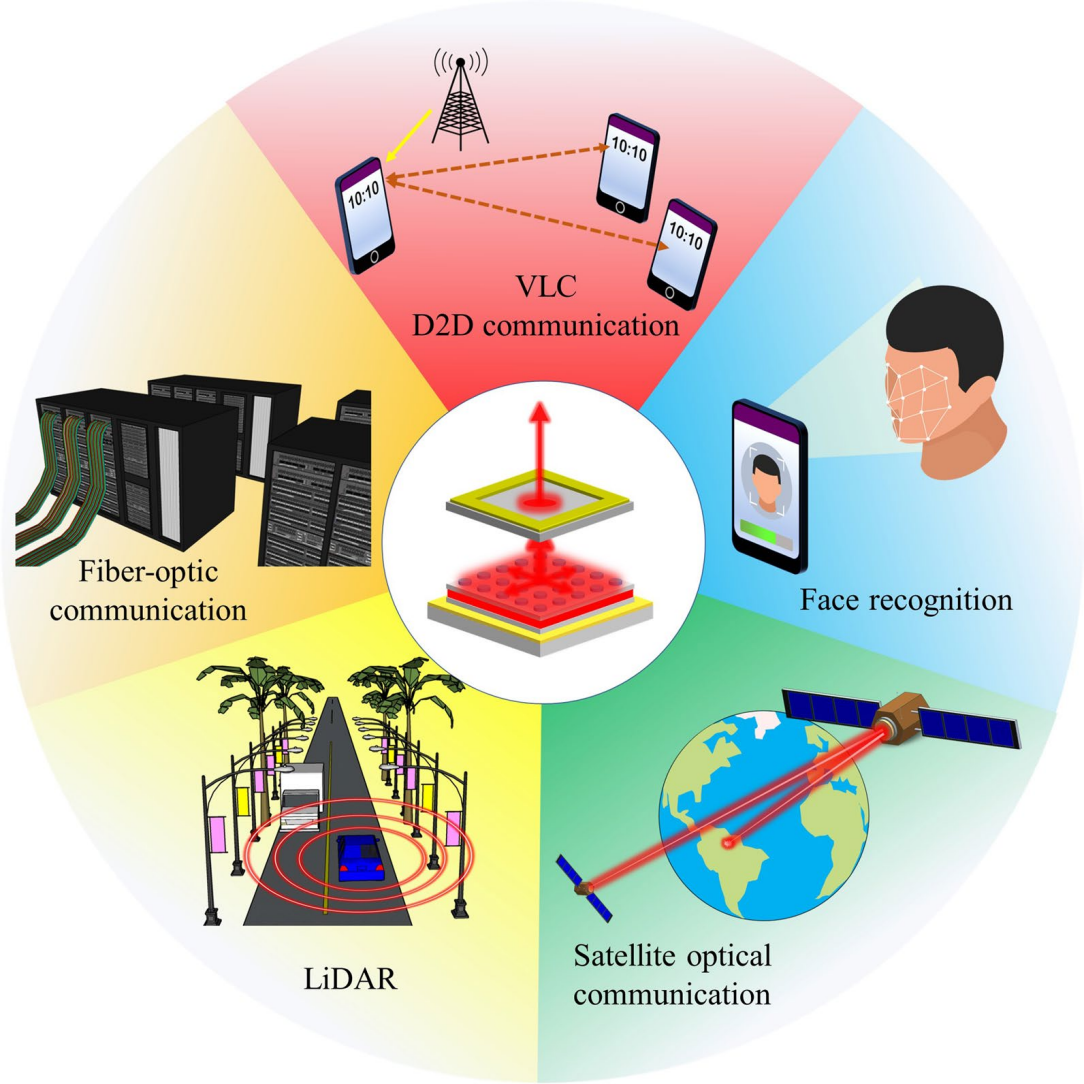
Schematic illustrations of PCSELs in miscellaneous scenarios

For laser optics, different application scenarios not only need different laser light intensity but also require laser beam quality, as shown in Fig. 1. The PCSEL technology meets these on-demand requisites with outstanding performance, and the corresponding output light intensity can be manipulated by adjusting the emitting area size with different lattice designs [37–39]. Hence, PCSELs have drawn people's attention, serving as a promising candidate for the next-generation light sources.

So far, PCSELs have been applied in the scenarios of light detection and ranging (LiDAR) [40] and face recognition [41, 42]. Besides, due to the high-speed responses, semiconductor lasers have been widely used for optical communication in data centres and satellites [43, 44]. Soon, it is believed that more and more semiconductor lasers will be adopted in consumer electronics for free-space D2D communications and even for optical communication transmitted between buildings in free space as well [6, 45]. Apart from optical communication, LiDAR applications are the next big market, requiring extremely short pulses for a better resolution in distance measurement resolution. Thus, pulse coding for high-resolution range imaging at an improved refresh rate is proposed [46].

In this paper, comprehensive performance analyses regarding the modulation speed of a commercial PCSEL (Hamamatsu) were conducted via small-signal measurement and the bit-error-rate test (BERT). Thereby, for the first time, radio frequency (RF) characteristics of such a laser light apparatus are investigated. Systematic analyses in both experimental measurement and theoretical calculation are carried out. A parameterised model is established based on the small-signal theory of semiconductor lasers, providing a deep insight into the corresponding intrinsic responses. Afterward, a commercial PCSEL is utilised for practical measurement and discussions.

## High-Speed Laser Theory and Simulation

Generally, the rate equation for semiconductor lasers describes the time-dependent relationship between carrier and photon numbers. The parameters required for the rate equation can be obtained from the epitaxial structure of a semiconductor laser. The parameters contained in the rate equation include carrier lifetime, photon lifetime, optical confinement factor, and so on. Therefore, the relationship between the current and the corresponding light intensity can be described through the rate equation. Apart from the relationship described above, the frequency responses of a semiconductor laser can be derived from the rate equation, which is named the theory of small-signal response. The equation of small-signal response is also dependent on the epitaxial structures. Hence, a simple parameter can characterise the frequency response of a semiconductor laser with a complex optical cavity or an epitaxial structure. The small-signal response is also known as the intrinsic response, $H_{{{\text{instric}}}} \left( \omega \right)$, and can be presented as below [47]:

1 $$H_{{{\text{intrinsic}}}} \left( \omega \right) = \frac{{\omega_{{\text{r}}}^{2} }}{{\omega_{{\text{r}}}^{2} + j\omega {\Omega } - \omega^{2} }}$$

2 $$\omega_{{\text{r}}} = 2\pi f_{{\text{r}}}$$

3 $${\Omega } = \frac{1}{{\tau_{{\text{n}}} }} + \tau_{{\text{p}}} \omega_{{\text{r}}}^{2}$$

4 $$f_{{\text{r}}} = \frac{1}{2\pi }\sqrt {\frac{{{\Gamma }v_{{\text{g}}} a}}{{eV_{{\text{a}}} }}\eta_{{\text{i}}} \left( {I - I_{{{\text{th}}}} } \right)}$$

where $\omega$ is the angular frequency, $f_{{\text{r}}}$ and $\omega_{{\text{r}}}$ are the relaxation frequency and its angular counterpart, respectively, and $\Omega$ is the damping factor corresponding to the intrinsic response. The damping factor ${\Omega }$ is affected by the carrier lifetime, $\tau_{n}$, a photon lifetime, $\tau_{p}$, and the angular relaxation frequency $\omega_{{\text{r}}}$. The relaxation frequency $f_{{\text{r}}}$ is influenced by the optical confinement factor, ${\Gamma }$, the group velocity of the semiconductor laser, $v_{{\text{g}}}$, the differential gain of the active region, $a$, elementary charge, $e$, the volume of the active region, $V_{{\text{a}}}$, injection efficiency, $\eta_{{\text{i}}}$, injection current, $I$, and threshold current, $I_{{{\text{th}}}}$. The relaxation frequency depends on the injection current and threshold current which are determined by the epitaxial structures and device layouts. The relationship between the modulation frequency and intrinsic response of a laser can be compared with Eq. (1) at different bias currents. The above formula provides the designer with a more intuitive way to evaluate the bandwidth of the laser.

Summing up the discussion above, if a laser cavity design can provide a high confinement factor and a small active region, the intrinsic bandwidth can be further ameliorated. Auspiciously, PCSELs can meet these on-demand requisites. The vertical laser light emission via the band-edge resonance of photonic-crystal structures with a high confinement factor can be realised in PCSELs. A small laser light emission region can be achieved via a double-lattice structure [48]. Such a high confinement factor is still unattainable for VCSELs. Therefore, to further discuss the effects of the confinement factor on the bandwidth for a PCSEL, we utilise the parameters of a high-slope efficiency PCSEL with a large emission area in Table 1 for demonstrations [47, 49]. To meet the design requirements of high-speed lasers, the emitter area of the PCSEL is set at a round emitter of 10 µm in diameter. The threshold current density of the PCSEL is set at 0.636 kA/cm^2^ according to the performance of the PCSEL with a large emission area [49]. Based on the above conditions, Eq. (1) can describe the intrinsic response of a high-speed PCSEL at different bias currents.

**Table 1:** The parameters for calculating the intrinsic responses of a PCSEL

Symbol	Description	Value	Unit
$v_{{\text{g}}}$	Group velocity [49]	$8.575 \times 10^{9}$	cm/s
$V_{{\text{a}}}$	The volume of the active layer^a^	$2.35 \times 10^{ - 12}$	cm^3^
$a$	Differential gain of the active layer	${9} \times 10^{ - 16}$	cm^2^
$\eta_{{\text{i}}}$	Current injection efficiency [47]	0.8	NA
$\tau_{{\text{n}}}$	Carrier lifetime [49]	2	ns
$\tau_{{\text{p}}}$	Photon lifetime (Q factor: ~ 10^4^)	5	ps
$I_{{{\text{th}}}}$	Threshold current [49]	0.5	mA

^a^A round emitter of 10 µm in diameter and 30 nm in thickness

**Fig. 2:**
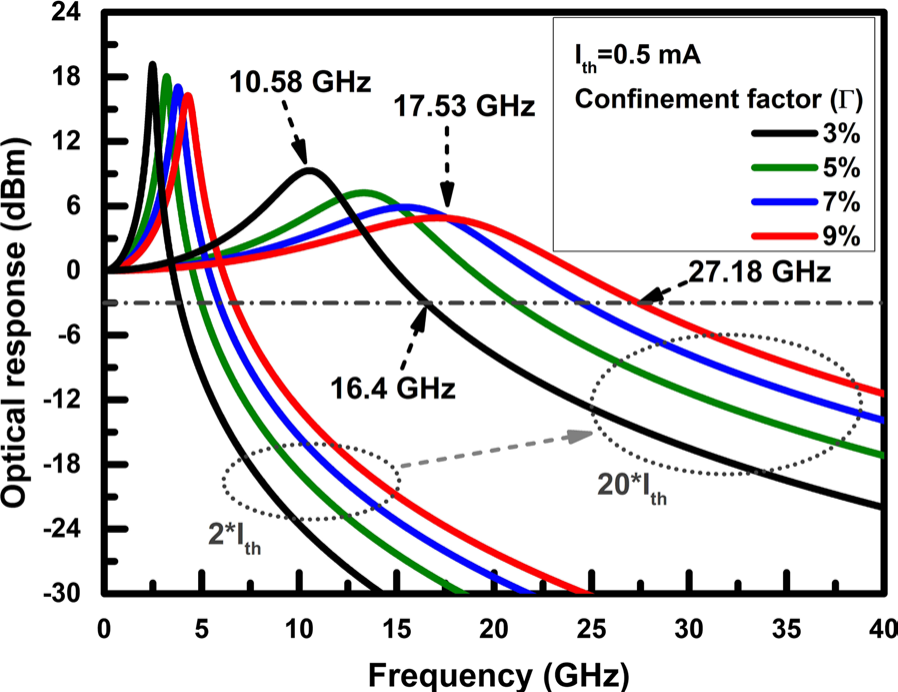
Intrinsic responses of a PCSEL biased at 2 and 20 times threshold currents, respectively. The corresponding confinement factors are varied from 3 to 9% at 2% intervals

Figure 2 shows the intrinsic responses of a PCSEL operating at 2 and 20 times the threshold currents, respectively. The corresponding confinement factors vary from 3 to 9% at 2% intervals. Such simulation results show that the bandwidth can be increased around 3–4 GHz when the optical confinement is increased every 2% at 20 times of threshold current. The relaxation frequency and the − 3 dB bandwidth rise from 10.58 GHz/16.4 GHz to 17.53 GHz/27.18 GHz when the confinement factor increase from 3 to 9%. Besides, the peak amplitude of frequency responses regarding the damping factor can be further reduced. When the confinement factors are raised from 3 to 9%, the peaking amplitude can be suppressed from 9 to 5 dBm at 20 times of threshold current due to the high relaxation frequency. Since a flat response is more conducive to the transmission of laser signals in general, the simulation described above confirms that improving the confinement factor can be a feasible strategy for high-speed optical communication.

## Optical Characteristics of PCSEL and RF Testing Setups

### Optical Characteristics

**Fig. 3:**
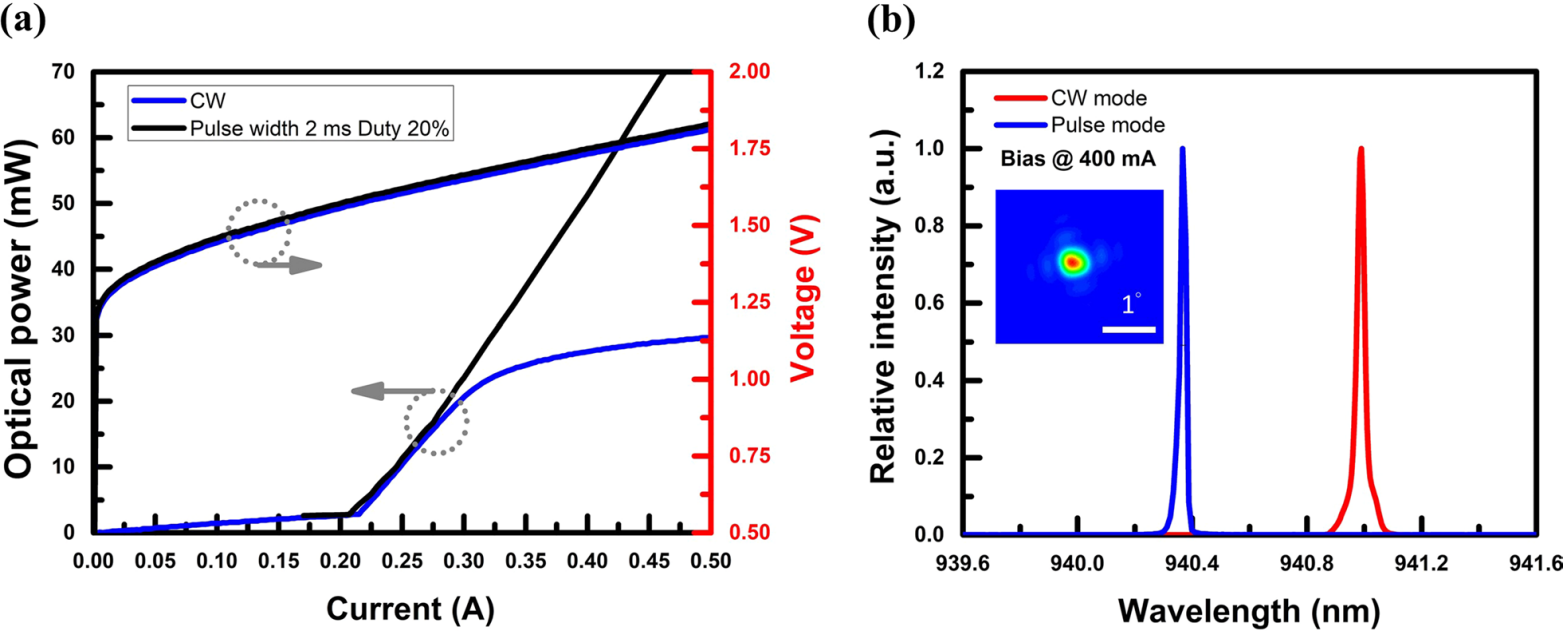
Optical characteristics **a** Light versus current versus voltage (L–I–V) curves of CW and QCW modes, **b** Optical spectra biased at 400 mA, and the inset shows a far-field image of the PCSEL operated in QCW mode

A commercial PCSEL (Hamamatsu) is employed to perform optical measurements, as shown in Fig. 3. The emitter area size is 200 × 200 in square micrometres. Such a PCSEL is operated in quasi-continuous-wave (QCW) mode and continuous-wave (CW) mode, respectively. In QCW mode, a pulse width of 2 ms with a 20% duty cycle is utilised. In this condition, PCSEL exhibits a threshold current of 215 mA. As shown in Fig. 3a, the corresponding slope efficiency and the differential resistance can be estimated at 0.286 W/A and 1 Ω, respectively. The spectra comparison between QCW mode and CW mode biased at 400 mA are shown in Fig. 3b. Besides, the centre wavelength in QCW mode is 940.3 nm. The divergence angle can be evaluated through far-field measurement. Hence, a divergence angle of 0.3° can be observed in QCW mode, shown in the inset plot in Fig. 3b.

Furthermore, self-heating effects originating from device thermal accumulation are also observed in CW mode. The slope efficiency of such a packaged PCSEL will rapidly drop to 0.025 W/A once the bias current is operated beyond 275 mA. In addition, a red-shift of corresponding centre wavelengths from 940.4 nm to 941.0 nm can be discerned. If the red-shift rate of the PCSEL is about 0.05 nm/K [35], the junction temperature difference is 12 °C. The spectrum's full width at half maximum (FWHM) rising from 0.027 to 0.033 nm also demonstrated that the PCSEL has a high junction temperature in CW mode. The gain variation with temperature is faster than that of the refractive index, and consequently linewidth broadening factor is small for lower temperatures [50]. Therefore, the linewidth of the PCSEL spectrum in CW mode is larger than that of the PCSEL operating in QCW mode. Yet despite the self-heating effects of a PCSEL is more severe in CW mode in general. The spectra and the subsequent bandwidth tests can be used to confirm that the PCSEL is still operated in laser mode instead of in LED mode.

### RF experimental Setups

**Fig. 4:**
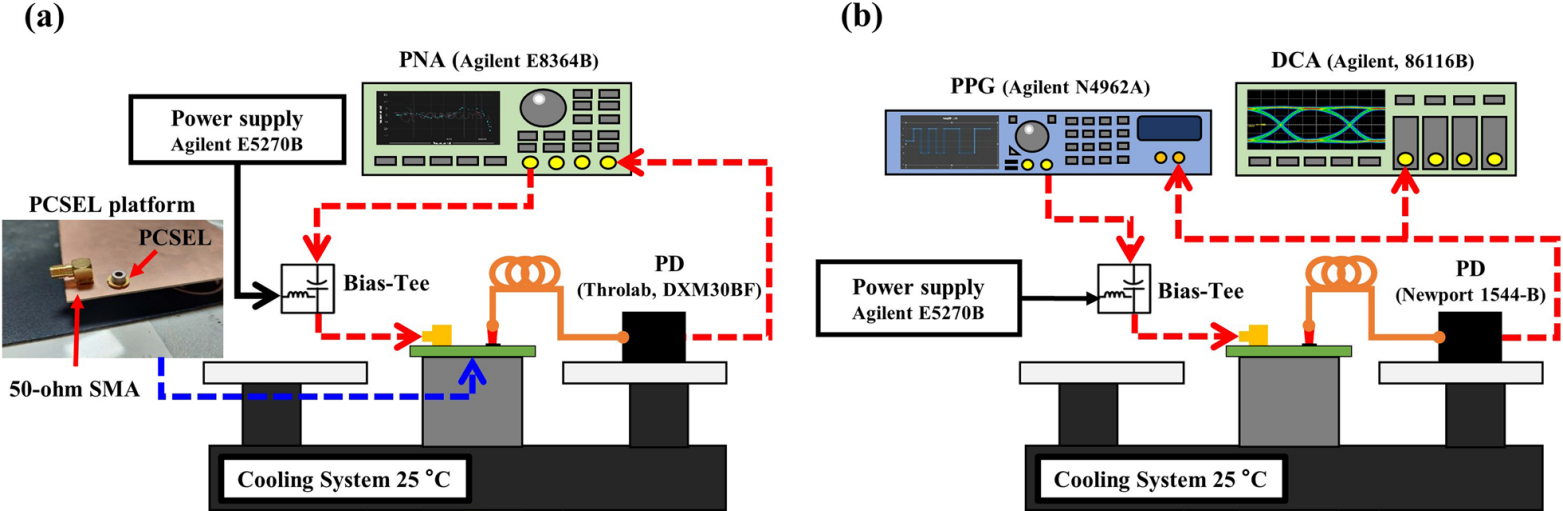
Experimental setups for **a** Small-signal response, and **b** BERT

The experimental setups of RF measurement for small-signal responses and bit-error-rate test (BERT) are shown in Fig. 4. A power network analyser (PNA, Agilent E8364B, 10 MHz to 50 GHz) is employed to discern small-signal responses and the testing frequency ranges from 100 MHz to 10 GHz with a small-signal amplitude of -5 dBm, as shown in Fig. 4a. The alternating currents (AC) and direct currents (DC) are coupled by a bias tee (Marki, BT-0065, from 4 kHz to 30 GHz). RF signals will be transmitted into the 50-Ω SubMiniature version A (SMA) connector via a high-speed cable. Subsequently, the optical signals from PCSEL are collected by a lens fibre (OM4, 3 m) and will be converted into electrical signals by a photodetector (Throlab, DXM30BF, DC-30 GHz). Finally, the electrical signals will feedback into the PNA, analysing the signal responses. Herein, the bandwidth of the photodetector is higher than the testing frequency, providing sufficient resolution. High-speed cables are de-embedded before such measurement. Therefore, the bandwidth of small-signal responses will mainly depend on the PCSEL and its package. All frequency responses from the PCSEL are obtained at different bias currents in CW mode.

After small-signal measurements, BERT with a non-return-to-zero on–off-keying (NRZ-OOK) signal is performed, as shown in Fig. 4b. Pseudo-random binary sequences are generated by a pulse pattern generator (PPG, Agilent N4962A), and the corresponding testing signals are transmitted into a 50-Ω SMA connector via a high-speed cable as well. Nonetheless, the signal reflection is unavoidable because of the impedance mismatch between the PCSEL and the SMA connector. This results in attenuation and interference effects on the optical signal. Thus, a high-speed photoreceiver (Newport 1544-B, DC-12 GHz) is adopted to convert optical signals into electrical signals. The electrical signals are subsequently analysed by a digital communications analyser (Agilent, 86116B). Finally, the bit-error-rate can be appraised by the PPG.

## RF Measurement and Analyses

### Bandwidth Measurement

**Fig. 5:**
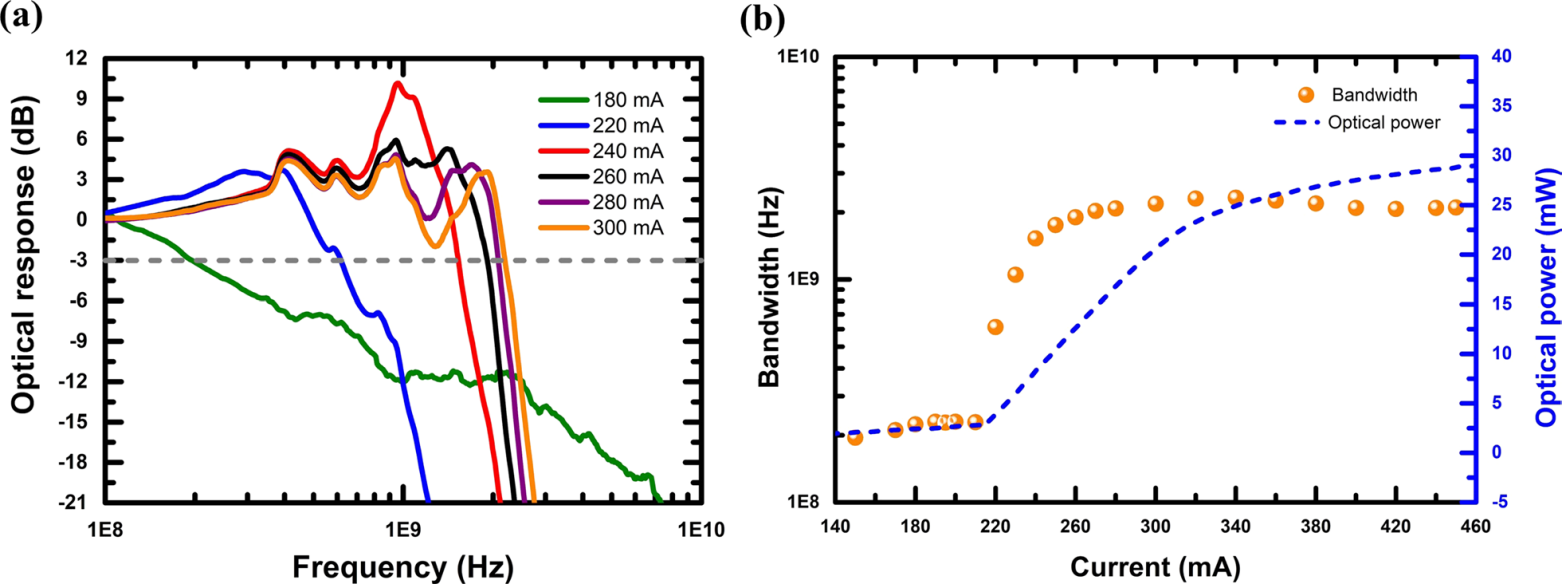
RF testing results of the PCSEL **a** Frequency response with distinct bias currents, **b** curves for bandwidths and the corresponding optical output powers versus different bias currents

The small-signal responses of PCSEL at different bias currents are shown in Fig. 5a. Thereby, the relationship between bandwidth, bias current, and the optical output power can be plotted in Fig. 5b. A saturated bandwidth of 229 MHz can be observed while operating below the lasing threshold of PCSEL. Once the bias current reaches the lasing threshold, spontaneous emission from PCSEL will switch to a strong stimulated emission, resulting in a rapid bandwidth extension. Moreover, while the bias current is over 280 mA, the bandwidth increasing rate will ease off, originating from the thermal effects, as shown in Fig. 5b. The thermal effects of bandwidth measurement can also be exploited with the optical output power versus bias current curve. When the bias current of PCSEL is up to 340 mA, a maximum bandwidth of 2.32 GHz can be identified. Eventually, due to the thermal accumulation, bandwidth saturation can be observed. The intrinsic bandwidth of a laser can be expressed not only by the current but also by the number of photons. Therefore, we can re-express Eq. (4) via the number of photons [47]:

5 $$f_{{\text{r}}} = \frac{1}{2\pi }\sqrt {\frac{{n_{{\text{p}}} }}{\tau }v_{{\text{g}}} a}$$

where $n_{{\text{p}}}$ is the photon density in a laser cavity, when the rising slope of the laser light intensity decreases, the number of photons no longer increases rapidly. Therefore, the resonance frequency cannot improve further, resulting in saturation of the − 3 dB bandwidth. It is worth mentioning that the small-signal bandwidth does not decay to the MHz level, indicating the lasing action from such a PCSEL does not downgrade to the spontaneous emission, namely operating in a LED mode.

### Relaxation Frequency Analyses

Typically, the total frequency response of a PCSEL should include the intrinsic and external parasitic responses. Thus, they can be extracted from the practical measurement afterward. But parasitic effects are often complex, including many parasitic capacitances, inductances, and impedance matching issues. As shown in Fig. 5a, several resonance valleys, namely at 530 MHz, 730 MHz, and 1.23 GHz, can be observed, indicating the influence of the parasitic effects cannot be ignored. Hence, to accurately extract the intrinsic bandwidth of a PCSEL, an equivalent RLC circuit is used to represent the external parasitic circuit. The total frequency response can be represented as below:

6 $$H_{{{\text{total}}}} \left( w \right) = H_{{{\text{intrinsic}}}} \left( w \right) \times H_{{{\text{RLC}}}} \left( w \right)$$

7 $$H_{{{\text{RLC}}}} \left( w \right) = \frac{1/LC}{{\frac{1}{LC} + jw\frac{R}{L} - w^{2} }}$$

The total frequency response of the PCSEL $H_{{{\text{total}}}} \left( w \right)$ includes the intrinsic response $H_{{{\text{intrinsic}}}} \left( w \right)$ and an equivalent parasitic circuit $H_{{{\text{RLC}}}} \left( w \right)$. $H_{{{\text{RLC}}}} \left( w \right)$ is an equivalent RLC circuit response for the parasitic effects [51], represented by resistance, *R*, inductance, *L*, and capacitance, *C*, respectively.

**Fig. 6:**
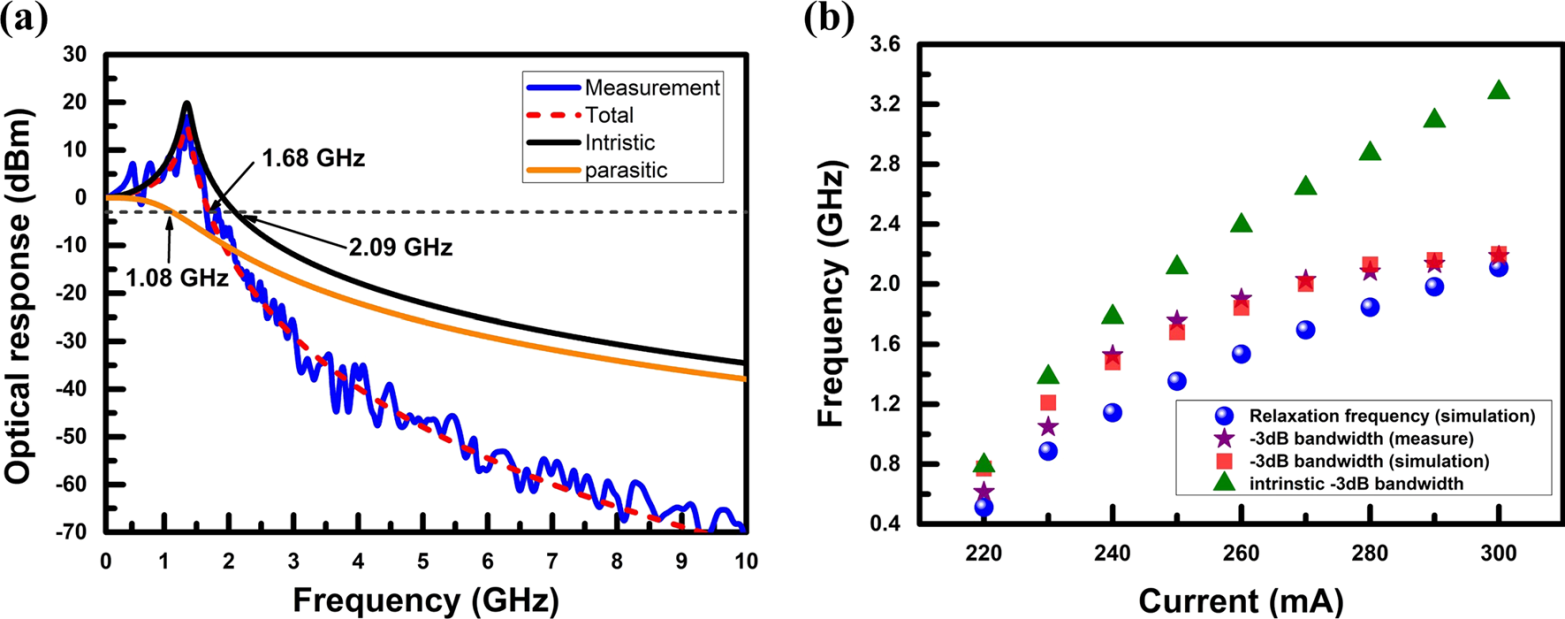
RF testing results and analyses: **a** optical frequency responses and the corresponding small-signal fitting with a bias current of 250 mA, **b** summarised bandwidth and the relaxation frequency from the calculation and practical small-signal tests

The parameters used to characterise the intrinsic response are shown in Table 1. However, proper modification is required based on the practical device. The active volume of PCSEL is assumed to be 230 × 230 × 0.03 in cubic micrometres, while the light-emitting area is assumed to be 200 × 200 in square micrometres. The length of the leakage current on one side is assumed to be 15 µm, and the active layer thickness is assumed to be 30 nm. The confinement factor is set to 8.5%. Thus, the intrinsic bandwidth of PCSEL can be calculated and extracted, as shown in Fig. 6a. While operating at 250 mA, the corresponding bandwidth will reduce from 2.09 to 1.68 GHz, originating from the external parasitic effects. The response bandwidth of the external circuit is only 1.06 GHz, severely limiting the bandwidth performance of PCSEL, attesting to the causes of resonance valleys in the small-signal measurement as well. To confirm the accuracy of intrinsic response calculation, we summarise the measured data at different bias currents in Fig. 6b. Before entering the thermal saturation region, the simulation results can fit the measurement results very well after considering external parasitic circuits. It can prove that the assumed parameters of the intrinsic response are credible. If a small active volume of the PCSEL can be realised, a high-speed PCSEL simulated in “High-Speed Laser Theory and Simulation” section has a chance to be achieved. The insight suggests that a high-speed PCSEL can be realised with a shrunk form factor [48].

Generally, the − 3 dB bandwidth is proportional to the relaxation frequency by f_−3 dB_ = 1.55f_r_ [47]. However, the − 3 dB bandwidth is gradually overtaken by the relaxation frequency when the operating current is increased, as shown in Fig. 6b. When the PCSEL is operated at 300 mA, the corresponding relaxation frequency is 2.11 GHz. The calculated − 3 dB bandwidth and practical measurements are 2.2 GHz and 2.18 GHz, respectively. The phenomenon indicates that the parasitic effects will suppress the − 3 dB bandwidth. To clarify which is the dominant factor of the PCSEL bandwidth at a given current density. The intrinsic bandwidth of the PCSEL is also added in Fig. 6b. The PCSEL extraction results show that the − 3 dB bandwidth gradually moves away from the intrinsic bandwidth with an increasing current. Although a high current helps to increase the intrinsic bandwidth, it still cannot compensate for the response loss caused by the parasitic bandwidth. The intrinsic bandwidth and the parasitic bandwidth will gradually reach a balance point when the current exceeds 260 mA so that the − 3 dB bandwidth of PCSEL tends to be saturated. Meanwhile, the thermal effect also slows down the rate of increase in the number of photons in the laser cavity. This phenomenon means that the intrinsic bandwidth no longer increases rapidly with current. Hence, the − 3 dB bandwidth of the PCSEL did not rise further with current. Once the issues of external parasitic effects can be resolved, PCSEL with the innate high confinement factor can achieve higher operating bandwidth.

### Bit-Error-Rate Test

**Fig. 7:**
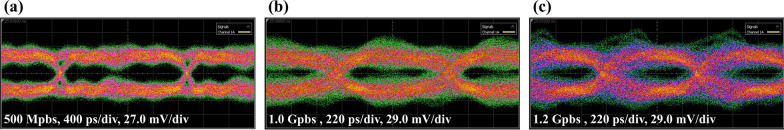
Eye diagrams of the PCSEL at **a** 500 Mbps, **b** 1.0 Gbps, and **c** 1.2 Gbps NRZ-OOK transmission in a back-to-back configuration (BtB, ~ 3 m-length fibre)

Through the BERT, the communication capability of PCSEL can be examined. Herein, the bias current of PCSEL is at 450 mA, driven by the NRZ-OOK signals with a peak-to-peak amplitude of 1.8 V. Test bit rates are 500 Mbps, 1.0 Gbps, and 1.2 Gbps, respectively. The maximum achievable data rate of 1.2 Gbit/s has a BER of 2.51 × 10^–3^, satisfying the FEC threshold of 3.8 × 10^–3^. There is a clear and open-eye diagram at 500 Mbps. At 1 Gbps, the eye is beginning to close and is virtually closed at 1.2 Gbps, as shown in Fig. 7. At the bit rate of 500 Mbps, the corresponding rise time of the eye diagram is 186.16 ps (10–90%) and the equivalent bandwidth can be acquired from the rise time. The relationship between rising time and equivalent bandwidth can be represented as below [52]:

8 $$- 3\,{\text{dB}}\, {\text{bandwidth}} = 0.35/{\text{Rise}}\,{\text{time}}$$

Thus, an equivalent bandwidth of 1.88 GHz can be obtained. While the PCSEL is biased at 450 mA, the small-signal measurement is 2.1 GHz, which is consistent with the equivalent bandwidth. A clearly open eye can be obtained at 500 Mbps. The eye-opening will gradually shrink while the transmission rate increases. The rising edge and falling edge of eye diagrams are split due to the resonance valleys of small-signal responses generated by the external parasitic effects. Eventually, the external parasitic effects will suppress the maximum bit rate at 1.2 Gbps.

## Conclusion

Comprehensive performance analyses of a PCSEL have been conducted via small-signal measurement and BERT. RF characteristics of a PCSEL are displayed for the first time. According to the intrinsic response simulation, the − 3 dB bandwidth of a laser can be significantly increased by a high confinement factor, confirming the impact of the confinement factor on the bandwidth. Thereby, a commercial PCSEL is exploited to examine the simulation results. The maximum bandwidth of the PCSEL is 2.32 GHz in practical. Based on the practical device, the parameters in the calculation are modified, leading to a good agreement with the measurement results. The results provide a promising perspective that a high-speed PCSEL can be realised with a shrunk form factor [48]. Moreover, transmission experiments are conducted as well. The maximum bit rate and corresponding rise time transmitted at 500 Mbps are 1.2 Gbps and 186.16 ps, respectively. Once the issues of external parasitic effects can be conquered, PCSEL with the innate high confinement factor can achieve higher operating bandwidth, serving as a promising candidate for the next-generation light sources in high-speed optical communication.

## Data Availability

Not applicable.
